# The Courage to Change the Rules: A Proposal for an Essential Health R&D Treaty

**DOI:** 10.1371/journal.pmed.0020014

**Published:** 2005-02-22

**Authors:** Nicoletta Dentico, Nathan Ford

## Abstract

The medical needs of many of the world's population go unmet. A new treaty on essential health R&D could provide a binding framework to redirect today's scientific expertise to priority needs

Biomedical science and technology are developing at a more rapid pace than ever. Investments in health research and development (R&D) have never been higher—global spending on health research increased from US$30 billion in 1990 to US$105.9 billion in 2001. But despite advances in technology and unparalleled research spending, the medical needs of many of the world's population go unmet. For example, only 1% of new drugs approved between 1975 and 1999 were specifically developed for tropical diseases and tuberculosis—diseases that account for over 10% of the global disease burden (Figure 1) [1].

**Figure 1:**
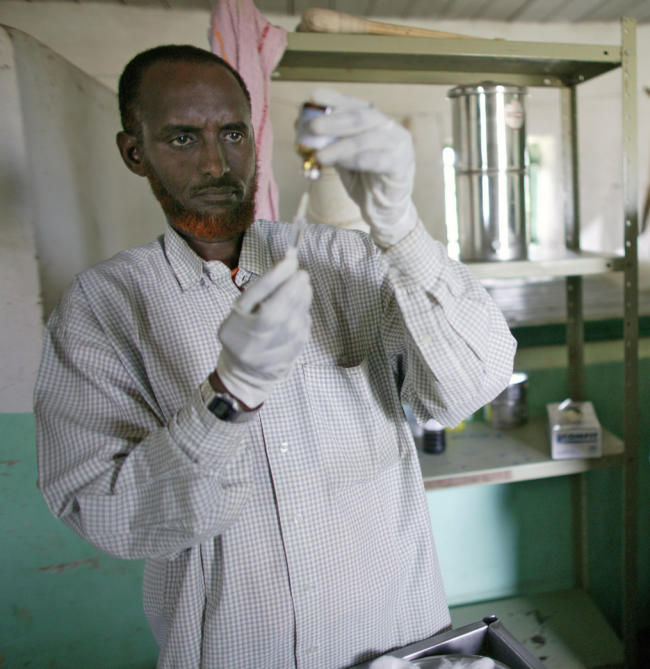
Clinical Officer Preparing Sodium Stibogluconate Solution Injection for a Patient with Visceral Leishmaniasis Sodium stibogluconate solution is administered by intramuscular injection for 30 days. The injection is painful and can cause toxic reactions. Developed in 1934, resistance of up to 65% has been documented in India. Around 50,000 people die from visceral leishmaniasis each year. New, effective drugs and diagnostics are urgently needed. (Photograph: Copyright Espen Rasmussen/MSF, Somalia, 2004)

In recent years, some important steps have been taken to improve access to existing treatments in the developing world by increasing generic competition. Yet there continues to be a tension between promoting access to lifesaving medicines as a human right and maintaining a global trade regime that seeks to finance health R&D by allowing monopolies to charge high prices [2].

There is a growing demand from many quarters for a new international policy framework [3]. A new international treaty on essential health R&D could provide a binding framework to redirect today's knowledge and scientific expertise to priority health needs. The treaty could help to cement new political commitments and coordinate complementary partnerships aimed at generating and rewarding health innovation as a global public good.

## The Patent System: An Unhealthy Motive for Medical Innovation?

Until recently, providing patent protection for pharmaceuticals was a choice made by individual governments according to their level of industrial development. Today, pharmaceutical patents are globalized through the World Trade Organization's Agreement on Trade-Related Aspects of Intellectual Property Rights (TRIPS Agreement) [4], and then further reinforced through bilateral and regional arrangements (the so-called TRIPS-Plus agreements [5]). But the patent system stimulates innovation only where industry sees the opportunity for increasing sales and market share; much of the resulting “innovation” is in fact imitation, producing “me-too” drugs that offer little, if anything, in the way of therapeutic benefit over existing drugs (Box 1).

**Box 1. How Innovative Is the Profit Motive?**

- A study published in the *Lancet* in 2002 showed that 68% of all new chemical entities marketed worldwide in the last 25 years were me-too products, representing little or no therapeutic gain [1].
- According to the United Nations Development Programme, less than 5% of drugs introduced by the top 25 pharmaceutical companies in the US represented true therapeutic advances; of these, 70% were developed with government involvement ([20], p. 69).
- Studies of drug development over the last decade in the US [21] and the last two decades in France [23] show that around two-thirds of medicines are me-too products.
- 92% of medicines approved in 2002 by the US Federal Drug Administration were me-too drugs [23].

The poorest are hardest hit. While R&D of new therapies against tropical diseases has ground to a standstill, 14 million people die from infectious diseases each year, predominantly in developing countries [1]. Most of the world's 40 million people with HIV/AIDS, including 2.2 million children under 15, live in the developing world (www.unaids.org). The poor also dominate non-communicable disease tables, accounting for 59% of the 56.5 million annual global deaths [6].

The patent system is also promoting new inequalities in high-income countries. Americans now spend a staggering $200 billion a year on prescription drugs. This figure is growing at a rate of about 12% per year [7]. The average price of the fifty drugs most used by senior citizens in America was nearly $1,500 for a year's supply in 2002. Prescription drugs have become inaccessible even to many people in the rich world.

Patents, with their focus on maximizing profits, have at least three negative consequences. First, it has been argued that the patent system causes substantial welfare losses because consumers who would buy the product if it were priced at somewhere nearer production cost do not buy it at the monopoly price [8]. Second, the system encourages counterfeiting—counterfeit drugs may represent up to 10% of the global market for pharmaceuticals [9]. Third, patented drugs are promoted through excessive marketing—on average, twice as much is spent on marketing a drug as on its R&D [10]. Across industries, it is becoming increasingly apparent that the patent system isn't working well [11], leading some in industry to express public concern that the blockbuster business model is “irreparably broken” [12]. A new approach is needed, for all our sakes.

## Prescriptions for an Innovative Approach: A New Treaty for Essential Health R&D

The only major international policy instrument that exists today to stimulate and finance health R&D is the TRIPS Agreement [4]. The TRIPS Agreement provides 20 years of patent protection on pharmaceuticals in the hope of stimulating the development of new medicines. Beyond that, governments try to stimulate R&D in neglected areas by providing industry with incentives such as tax breaks and patent extensions. However, the effectiveness of these policies is hardly known.

In 2001, the Doha Declaration on TRIPS and Public Health affirmed the sovereign right of WTO members to take measures to protect public health by overcoming patents whenever needed [4]. The last few years have seen increased attention to the fact that patents keep drug costs high and limit access to medicines. However, there has been no movement in international policy to address the crisis in pharmaceutical innovation.

Health R&D must be treated as an international problem that requires an international solution. It should be treated like other strategic sectors, as happens today for defense and space discovery—sectors that both benefit from very strong government support for innovation. When global public goods do correspond to national needs, governments should step in to mobilize and enforce the collective action required. For example, global cooperation in the sharing of infectious disease monitoring from 1890 onwards set a valuable precedent [13].

The recent epidemic of severe acute respiratory syndrome—SARS—clearly shows that biomedical knowledge and the pharmaceutical sciences can be mobilized to achieve rapid advances relevant to social needs if sufficient resources and political will can be mustered. The SARS virus was completely sequenced in just six days, and a diagnostic test was developed in only three months. The public-sector funded, collaborative “public-goods model” used for the Human Genome Project shows that public collaborative research can be more efficient than the closed, monopolistic, private sector approach.

An international treaty (Box 2) would promote a health-needs-driven approach to drug discovery. The elaboration of such a treaty would have to meet the two crucial requirements for an effective system of funding innovation in pharmaceuticals. First, the reward for innovation should be proportional to the social (that is, therapeutic) value. Second, prices should be near average production cost.

**Box 2. Key Concepts of an R&D Treaty**

**A global, needs-driven R&D agenda:** allowing policy makers, funding agencies, and the research community to set priorities for developing safe, effective, and affordable medicines according to health needs.

**Prioritization for neglected diseases:** to ensure that immediate efforts are made toward finding new tools for lethal diseases that are currently difficult or impossible to diagnose and treat.

**Adequate international financing of health R&D:** a new funding mechanism is urgently needed to support R&D on an ongoing basis, particularly for neglected diseases. All governments will need to participate according to their means.

**Equitable pricing:** governments should ensure that the poor also have access to innovations resulting from government-funded or university research.

**Open access:** governments should require access to the compounds and tools that result from public research in order to stimulate follow-on innovation elsewhere.

**International exchange:** strengthening openness and transfer of technologies on a global basis will greatly help developing countries by improving access to information and ideas and accelerating the development of science and technology.

**Figure 2:**
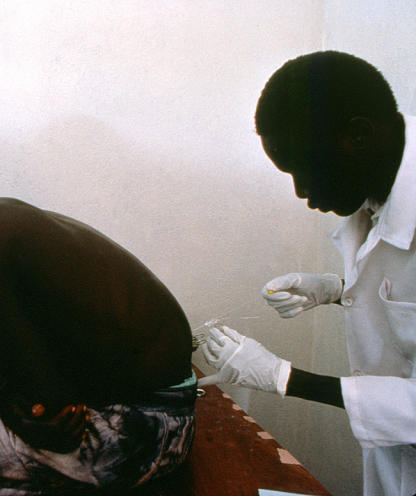
Detection of African Sleeping Sickness by Lumbar Puncture Lumbar puncture in patients with African sleeping sickness can be a painful and potentially dangerous maneuver that is the only way to determine if the disease has progressed to the second stage. New diagnostic tools are urgently needed, as are new treatments; current medicines are old, toxic, difficult to use, and their production is not guaranteed. Around 60,000 people die from Afican sleeping sickness every year. (Photograph: copyright Serge Sibert/MSF, Uganda, 1998.)

The idea is to shift the discourse from trade to health. The treaty—focussed directly on R&D rather than patent rights or drug prices—would address the global management of publicly funded health R&D. Priorities for R&D would be defined through public-sector leadership and based on public health needs. R&D opportunities would be aimed at new lead compounds, new types of health tools (Figure 2) and new treatment approaches. As the only legally mandated international government agency responsible for global health, the World Health Organization should work toward establishing this essential R&D agenda. Individual states would need to periodically evaluate targets for priority research and make adequate recommendations toward needs-driven R&D.

## How Will the Treaty Work?

One of the main objectives of the treaty would be to encourage the broad dissemination of information and knowledge-sharing, and to support diversity, competition, and collaboration among researchers from developed and developing countries.

There are already precedents for the free, public sharing of innovations with the aim of developing new drugs. The Tropical Diseases Initiative (www.tropicaldisease.org), for example, is a new, Internet-based, community-wide effort to develop new drugs for tropical diseases [14]. The BioBricks project (http://parts.mit.edu/) at the Massachusetts Institute of Technology is exploring standardized tools and processes for DNA work, largely by computer. The Bios Initiative (Biological Innovation for Open Society), launched by the Australian non-profit organization Cambia (the Center for the Application of Molecular Biology to International Agriculture; www.cambia.org), is an effort to develop new innovation systems for market failures and for neglected priorities [15].

Among other incentives, technology exchange frameworks could include licensing agreements with developing countries, or affirmative commitments of research funds for collaborative projects with these countries. Such collaboration is currently being implemented in Europe, for example, through the European and Developing Countries Clinical Trial Partnership (www.edctp.org) [16]. The partnership is a new funding body established to fund research in developing countries, particularly in Africa, which contributes to the development of affordable prophylactics and drugs for HIV/AIDS, tuberculosis, and malaria. The treaty on health R&D should also promote partnerships between countries in the developing world and encourage the creation of regional technology networks in developing countries.

Much of today's drug development know-how exists within the private sector. Further work is needed to define obligations and incentives in the treaty that maximize industry contributions to publicly funded R&D by providing in-kind contributions in areas where industry has the skills that public groups need. The treaty should also provide an expanded use of government rights against patent abuse on drugs developed with public support. This would include the right of a government to intervene if an invention is not made available to the public on reasonable terms, such as is included in the march-in rights clause of the United States's 1980 Bayh-Dole Act (which enabled public universities to license inventions for commercial development [17]).

## Making the Treaty Happen

There are a number of obvious difficulties in moving the treaty forward, and these should not be underestimated. A delicate issue is the treaty's relation with other binding agreements, particularly the TRIPS Agreement. Governments that join the treaty should be granted patent exceptions and should not be accused of “free-riding,” since they would be contributing to R&D through a different juridical avenue.

Substantial government resources would need to be mobilized to finance the highest priority medical research. All governments should participate according to their means. Countries already contribute significantly to global R&D through the purchase of costly patented drugs. Among other measures, not-for-profit initiatives working to develop new drugs, vaccines, and diagnostic tools for neglected diseases should be funded at levels that enable them to reach their objectives. Recent examples clearly show that when political will is mobilized, resources are rapidly made available to generate R&D in a particular area. In 2001, the anthrax scare in the US led to increases in biodefense research spending at the US National Institutes of Health from US$53 million in 2001 to US$1.6 billion in 2004.

A treaty on health R&D is certainly a feasible proposal—the successful adoption of a treaty on plant genetic resources shows that it can be done. After seven years of negotiations, the Food and Agriculture Organization of the United Nations adopted the International Treaty on Plant Genetic Resources for Food and Agriculture in November 2001 [18]. This legally binding treaty covers all plant genetic resources relevant for food and agriculture. Through the treaty, countries agree to establish an efficient, effective, and transparent multilateral system to facilitate access to plant genetic resources for food and agriculture, and to share the benefits in a fair and equitable way. While there has been a growing consensus in development circles that more international public goods need to be supplied as part of the development strategy, increasing their provision will be influenced by the extent to which inspirational groups of individuals step in to play a leadership role to meet the collective need.

The Ottawa Convention to Ban Anti-Personnel Landmines and the 2003 Framework Convention on Tobacco Control show that international frameworks are essential to the regulation of the private sector for the good of global health. The World Health Organization, together with other relevant United Nations agencies, has full legitimacy to work with member states toward crafting challenging proposals, and provoking policy action. One lesson from these treaties is that support will have to be built from a strong coalition of like-minded countries that would steer the process internationally.

While the development of the treaty is still at an early stage of discussion, the concept is already being aggressively opposed. As with tobacco, landmines, and more recently, sugar, the involvement of civil society will be crucial to defend these health improvement strategies where these may conflict with powerful vested interests in the private sector [19].

It takes courage to change the rules. If governments are indeed persuaded to face up to their responsibilities in the coming years, it may very well be because of the many voluntary organisations that seek to promote the global public interest.

## Abbreviations

R&D: research and development
TRIPS Agreement: the World Trade Organization's Agreement on Trade-Related Aspects of Intellectual Property Rights
